# The Ideal Diet for Humans to Sustainably Feed The Growing Population – Review, Meta-Analyses, and Policies for Change

**DOI:** 10.12688/f1000research.73470.1

**Published:** 2021-11-10

**Authors:** Galit Goldfarb, Yaron Sela

**Affiliations:** 1 Nutrition, OUS University, The Royal Academy of Economics and Technology, Zürich, Switzerland

**Keywords:** Optimal nutrition, optimal diet, diet for health, early human diet, human evolution and diet, nutrition, nutrition and disease, diet for health, nutrition for health

## Abstract

INTRODUCTION:

As of now, no study has combined research from different sciences to determine the most suitable diet for humans. This issue is urgent due to the predicted population growth, the effect of this on the environment, and the deterioration of human health and associated costs.

METHODS:

A literature review determined whether an optimal diet for humans exists and what such a diet is, followed by six meta-analyses. The standard criteria for conducting meta-analyses of observational studies were followed. A review of literature reporting Hazard Ratios with a 95% confidence interval for red meat intake, dairy intake, plant-based diet, fiber intake, and serum IGF-1 levels were extracted to calculate effect sizes.

RESULTS:

Results calculated using NCSS software show that high meat consumption increases mortality probability by 18% on average and increases diabetes risk by 50%. Plant-based and high-fiber diets decrease mortality by 15% and 20% respectively ( *p* < .001). Plant-based diets decreased diabetes risk by 27%, and dairy consumption (measured by increased IGF-1 levels) increased cancer probability by 48% ( *p* < 0.01). A vegetarian or Mediterranean diet was not found to decrease the probability of heart disease. A vegetarian diet can be healthy or not, depending on the foods consumed. A Mediterranean diet with high quantities of meat and dairy products will not produce the health effects desired. The main limitations of the study were that observational studies were heterogeneous and limited by potential confounders.

DISCUSSION:

The literature and meta-analyses point to an optimal diet for humans that has followed our species from the beginnings of humankind. The optimal diet is a whole food, high fiber, low-fat, 90+% plant-based diet. This diet allowed humans to become the most developed species on Earth.

To ensure people’s nutritional needs are met healthily and sustainably, governmental dietary interventions are necessary.

## Introduction

As of now, there has not been a study that combines research from all of the available sciences to conclude what is the most suitable diet for humans that can heal and also prevent chronic disease and at the same time be sustainable for the environment. It is essential to address this issue urgently due to the predicted growing human population, increasing healthcare costs, and lessening agricultural land to grow foods due to global warming.

This research aims to determine whether there is one ideal diet for all humans. And if so, to examine how individuals and governments can use this knowledge to create policies that ensure the growing global population is fed healthily and sustainably without causing further environmental destruction.

Research shows that 95% of the global population is living in poor health. ^1^^–^^3^ The obesity epidemic has led to higher incidences of noncommunicable diseases (NCDs) including heart disease, type 2 diabetes, and specific cancers. ^4^^–^^9^

Currently, individuals are confused as to what diet is ideal for promoting health and longevity.

Individuals need to have a set standard for healthy food based on human needs, to enable them to improve health and require less healthcare in the future.

An extensive literature review combining data from different scientific fields helped determine underlying factors relating to the ideal diet for humans. A look at human evolutionary research was necessary for a thorough approach to this subject.

The literature review points to a major change within the hominin lineage ( *Australopithecus)* occurring about 5 million years ago with the evolution of bipedalism.

This locomotive change came to hominin benefit about 2 million years ago, following a global change in weather conditions when the earth entered the Ice Age.

The North African tropical rain forest began receding and the savanna grasslands expanded. Bipedalism gave hominins the ability to leave the shrinking rainforest where food resources were dwindling as a result of the ice age, for the growing savannas. ^10^ Skull fossils show that hominins living in the rainforest had a brain size of 320-380 cm ^3^ in volume. ^11^^–^^13^

Upon moving to the savanna grasslands, hominins needed to change dietary practices to survive. Herbaceous leaves that were plentiful in the rain forests were sparse on the savanna, forcing hominins to start feeding on other foods. Dental microwear and stable isotope analysis show evidence of C4 resources, mainly underground storage organs (USOs) (tubers, roots, and bulbs), as a significant component of hominin diets on the savanna. ^14^^–^^22^

The next hominin fossils found of the genus *Homo, Homo habilis*, demonstrate a steady process of brain growth to 400-600 cc.

The dry savanna habitat was also rich in sedge and grass grains, rich in carbohydrates, perfect to nutritionally support hominin brain growth. ^20^^–^^28^

A larger brain requires more energy to fuel. It is a metabolically expensive organ, and therefore requires a stable, high-energy, nutrient-dense food source to support its growth under natural selection. ^16^^,^^20^^,^^29^

Fossils of the next ancestor of the genus *Homo*, *Homo ergaster,* who lived exclusively on the savanna, show a brain size 50% larger than their predecessor *Homo habilis* (800–1200 cc). ^30^^,^^31^ The larger more advanced brain allowed *Homo ergaster* to thrive in the hostile savanna environment. *Homo ergaster* had a jaw and tooth size closely resembling that of modern humans, and fossils show that their digestive tracts grew smaller.

Due to the vast number of animals on the savanna, it would seem obvious to suggest that the change in diet was towards a meat-based diet, however, to be dependent on meat as a stable food source on the savanna, hominins would have needed to hunt big game that roamed the savanna. To hunt successfully, hominins would have needed to master the skills of hunting. Research suggests that hunting skills have a very long learning curve. ^32^^–^^34^

Evidence also suggests that big game roaming the savanna were a poor food source for hominins. Their meat was lean, with almost no fat, and the protein levels were too high to consume in abundance, rendering them an inefficient source of calories.

Dietary protein, when consumed in excess, becomes toxic to the human body **.** Humans can metabolize about 250 grams of protein per day; exceeding this level produces toxic waste that the body has difficulty eliminating. Furthermore, some of the protein consumed is required for cellular growth and repair and would not be available for energy. In fact, 250g of protein provides only 1000 calories, not nearly enough to sustain a modern-day sedentary adult, let alone an active hunter-gatherer on the harsh African savanna. It is well documented that those who consume excessive amounts of lean protein, without sufficient fat, develop a condition called “mal de caribou,” ^35^ whereby ammonia builds up in the blood. If this diet persists, the person will suffer from diarrhea and mineral losses and will eventually die. ^36^^–^^39^

Research shows that modern hunter-gatherer groups such as the Hadza and San, with modern sized brains, fail to catch meat on 97% of their hunts, and share the meat mainly with their co-hunters. Furthermore, hunting is usually practiced when staple foods are available in abundance. ^40^^–^^46^ It is suggested that hunting occurred primarily for rituals and shows of courage rather than solely for dietary necessity. ^46^^–^^48^

In 1993, more than 1,000 researchers participated in a study titled *The Lost Crops of Africa*, examining ancient crops of Africa. The report comes to a clear conclusion: “Grass seeds have sustained humans throughout time.” ^49^^,^^50^

In 1984, anthropologists found the nearly complete skeleton of a *Homo ergaster* child assumed to be 1.7 million years old. The skeleton, referred to as Nariokotome Boy, ^51^^,^^52^ had a brain size of 880 cm ^3^ in volume. In the fossil remains we see evidence from the shape of the ribcage, that at this stage, hominin gut size shrunk to the size of a modern human gut. This shrinkage of gut size allowed the available energy to be directed to feed the growing brain.

The brain is an incredibly energy expensive organ using 22% of human basal metabolic energy. To accommodate the increase in brain size, humanity’s forebears needed a good, high-quality, readily available food source. ^53^

Such food sources grew readily all year round on the savanna and include legumes, grains, plants with USOs, seeds and fruits. ^54^^–^^76^

The switch to starchy foods, very different from the dietary habits in the rainforests, allowed early humans to thrive on the savanna with shorter guts which required less energy to maintain. The excess energy became available for brain tissue growth ^77^ because hominin survival depended on intelligence.

Enzyme inhibitors in plants stop enzymatic reactions. As a result, enzyme inhibitors can have an anti-nutritional effect. ^77^^–^^79^

Cooking deactivates these anti-nutrients. But when cooking was still unavailable, anti-nutrients could easily be deactivated by a simple soaking in water ^61^^,^^79^^,^^80^ causing enzyme inhibitors to stop functioning. Also, an adapted gut microbiota helps the breakdown of such starches. ^79^ Furthermore, young grass grains do not have enzyme inhibitors.

The probable low bio-accessibility of nutrients from legumes and grains is less relevant due to the wealth of legumes, grains and tubers available on the savanna. ^79^^–^^83^

USOs, by contrast, have a physical defense mechanism by being located underground or covered in thick outer layer. USO-bearing plants are edible in raw form. ^74^

Fossil dental calculus, accepted as a significant pool of dietary data, show that Neanderthals 400,000 years ago ate a wide variety of plant matter especially USOs and grass seeds. Neither geographic region, species, nor known stone tool technology had a significant impact on the number of plant species consumed. ^84^ Fossilized Neanderthal feces was also found to have large amounts of plant matter. ^85^ Other research has also revealed that plant foods had a leading role in early human diets. ^84^^,^^86^^–^^91^ In later fossils, evidence of damaged grass seeds in dental calculus are a sign of cooking. ^84^^,^^92^^–^^94^

The control of fire by hominins began sometime between 400,000-700,000 years ago. At this time, another major brain growth spurt in hominin fossils is observed (from 800 cm ^3^ to 1,100 cm ^3^ in volume). ^86^^,^^95^^–^^100^ Fire enabled a reduction in the need for chewing and detoxification of anti-nutrients, making more energy and nutrients available for the brain and the body. ^101^^,^^102^

Approximately 195,000 (±100,000) years ago evidence shows that the first *Homo sapiens* (modern humans) appeared and replaced other *Homo* species in Africa. Omo, the oldest fossil remains of modern humans, show that they had the same anatomical build as we have today. ^103^^,^^104^

12,000 years ago, agriculture began at the geographic corridor through which humans left Africa. The first foods chosen to be grown through agriculture were grains, emphasizing their significance for humans during the hunting-gathering period.

Grains and legumes were easily domesticated from their wild ancestors because they required very little genetic change to domesticate. ^105^

Animals were domesticated 6000 years ago only in a few areas on earth, principally in Asia, and Europe. Animals were rarely domesticated in America. Animals were ***not*** domesticated in tropical Africa or Australia. ^106^^–^^108^ This lack of domestication is probably due to the abundance and variety of grains, legumes and USOs found in these places, reducing the need to domesticate animals for food.

The domestication of animals for food resulted in increased meat consumption beyond previous consumption patterns of hunter-gatherers who ate meat sparsely when available. ^109^^–^^112^

Human brain size decreased after the dawn of agriculture. ^112^^,^^113^

Previous to agriculture, life expectancy was past the age of menopause in women. When agriculture was introduced, life expectancy dropped to 40 years because human eating habits changed dramatically. ^112^^,^^114^ Only certain crops were grown, exposing farmers to many risks and leading people to suffer from severe nutrient deficiencies, shortening lifespans. ^105^^,^^112^^,^^115^^–^^117^

After the industrial revolution, life expectancy increased, but quality of life did not necessarily follow suit. ^118^^–^^124^

The awe for meat and processed grain consumption increased with the industrial revolution when food processing became popular for storage and transport. Grains that could originally supply a wealth of nutrients, were stripped of their healthy bran and germ layers becoming nutritionally deplete. This move to processed grains caused large populations to develop nutrient deficiencies including protein deficiency which led to the discovery of a disease named Kwashiorkor. Kwashiorkor was caused by consumption of nutrient and protein depleted dried grains that were ground into flour for children as food. Kwashiorkor sometimes healed with animal protein consumption. ^125^^,^^126^

However, nutrient and protein shortage were never a problem for humans until grain processing began. ^127^^–^^130^

A whole-food, mostly plant-based diet, from varied plant sources, as ancient humans consumed, easily incorporates all of the nine essential amino acids for humans.

Protein content in human breast milk is lowest in comparison with other lactating mammals and is higher in carbohydrates, and mono- and polyunsaturated fats. ^131^^,^^132^

This suggests that although protein is necessary for human health, consuming large quantities of protein carries a considerable price on human health.

In recent decades, the world has seen a rise in NCDs such as heart disease, cancer, and diabetes. Seven out of every ten deaths are due to NCDs. ^133^

Nowadays domesticated animals are rich in fat. The fatty deposits among muscle fibers soften the cooked meat and improve their flavor.

The fat composition of domesticated meat has also changed over time. Previously, animal fat had equal amounts of ω-6 and ω-3 fatty acids (FAs). Nowadays animal fat is rich in inflammatory ω-6 FAs and low in health promoting ω-3 FAs because of the intensive rearing methods. ^134^^,^^135^ The levels of ω-3 FAs in meats 100 years ago were 170 mg/100 g of meat. Now they are 20 mg/100 g of meat. ^134^^,^^136^

Animal milk is also less suitable for human consumption. Milking animals only began around 6000 years ago. ^137^

Most of the current world population (75%) is lactose intolerant, leading to side effects such as mineral losses, diarrhea, cramping, bloating, and gas.

It is also common for only about 25% of dairy calcium from milk to be absorbed. The remaining unabsorbed 75% may end up deposited around the body, leading to atherosclerosis, gout and kidney stones.

## Methods

A will to bring to the table valid results that prove the literature review formed the reasoning behind the following meta-analyses, since meta-analysis is considered by many to be the platinum standard of evidence.

A look at mortality statistics for people following different diets as well as a look at different dietary patterns and the most common diseases in the world today (cancer, heart disease and diabetes, were examined through a collection of studies performed in the last decade only. The results were calculated using NCSS 2019 software available for free at: https://www.ncss.com/download/ncss/free-trial/. Furthermore, other meta-analyses that were performed in this time period were used as a comparison between results received with the meta-analysis results received in this research.

### Search strategy

The standard criteria for conducting and reporting of meta-analyses of observational studies was followed (Stroup et al, 2000). Studies were identified through a systematic review of the literature by using the PubMed and Google Scholar databases against a list of pre-defined comprehensive search terms on 17 March 2019 and updated on 28 March 2019 to search for more recent papers. Searches were run independently in each database because of their different set-ups, different thesaurus terms such as Medical Subject Headings (MeSH), and other relevant subject heading searching, and keywords. Exclusion criteria were (a) reviews, protocols, conference abstracts, practice guidelines, opinions, discussion pieces, editorials, commentaries, book chapters, and case reviews. (b) No limits were applied for language and foreign papers were translated (c) publication dates before December 2008, and after December 2018, except meta-analyses which were accepted.

Seven different meta-analyses combining the results of multiple studies were performed to support the argument that this ideal diet for humans can prove itself as the optimal diet also in our day and age. Each meta-analysis had its own hypothesis. The probability factor (p = value) was calculated to see if the null hypothesis was rejected or not. If the probability was small (less than 5% or less than 1 in 20 chance of being wrong), then the null hypothesis was rejected, and I could safely conclude that there was a connection between the independent explanatory variable and the dependent variable.

The search terms used for all studies included keywords, and subject headings, for example “meat,” “beef,” “red meat”, processed meat”, “unprocessed red meat”, “pork,” “veal,” “lamb,” “steak,” “hamburger,” “ham,” “bacon,” or “sausage,” “IGF-1,” “dairy,” “milk,” “Plant-based,” “vegan,” “vegetarian,” “fiber,” “Mediterranean diet,” “healthy diet” in combination with “mortality,” or “death”, or “heart disease,” or “diabetes,” or “cancer.” In addition, the reference lists of relevant publications were also searched for more studies. The search is archived at: https://doi.org/10.17605/OSF.IO/64NAM.

### Study selection

Papers were selected if they met the followed inclusion criteria (a) Prospective studies performed only in the last ten years, between December 2008 and December 2018 (as required by OUS university), (b) studies that reported relative risks and Hazard Ratios with 95% confidence intervals for the associations of unprocessed red meat, processed meat, total red meat consumption, dairy, high fiber, plant-based, vegan, vegetarian, with all-cause mortality, diabetes, cancer and heart disease. (c) Meta-analyses that were performed in the last ten years but included also studies performed from beforehand, were added to each meta-analysis performed for every subject so that the aggregated HR derived from the analysis could be compared and thus, the results would have a more holistic nature.

The search strategy retrieved 91 articles. After removing the duplicates, the title/abstract screening process identified 88 studies. After a further full-text assessment for sufficient statistics, 44 articles were excluded from the systematic review and 37 met the inclusion criteria for all studies, (please see search strategy in data availability section). No additional publications were found through reference lists and hand searching.

### Data extraction

From each publication, the first author's last name, year of publication, study location, gender, age, sample size (total number of participants and number of deaths or disease), relative risks and Hazard Ratios with a 95% confidence interval for each category of red meat intake, dairy intake, plant-based diet, fiber intake, serum IGF-1 levels, and covariates adjusted for in the analysis were extracted.

### Data analysis and statistical methods

The primary goal of meta-analysis was to compute the aggregative effect of specific food group consumption on mortality/disease, taking into consideration standalone and heterogeneous results. In order to examine the aggregate effect size, individual studies were gathered estimating mortality of people consuming a specific food group (e.g. red meat/high-fiber/plant-based), in comparison with people who do not consume this specific food group. Each individual study calculated Hazard Ratio (HR), the event rate corresponding to the conditions (dead/alive; disease/no disease) described by two levels of an explanatory variable (red meat vs. no red meat; vegetarian vs. non-vegetarian; high fiber vs. low fiber).

The main function of the meta-analysis was to estimate effects in the population by combining the effect sizes from a variety of studies. Specifically, the estimate is a weighted mean of the effect sizes. The ‘weight’ that is used is usually a value reflecting the sampling accuracy of the effect size, which is typically a function of sample size. The final goal of the meta-analysis was to determine the aggregative effect size beyond all effects that were gathered, its significance, 95% confidence interval and possible moderators for the results (variables that could explain non-random variance between effects).

For the final effect, the *HR* as effect-size estimate; a confidence interval (lower limit [LL] and upper limit [UL]); *Q* statistic; and its *p* value were reported. *Q,* a chi-square statistic, reflects variability among effect estimates due to true heterogeneity, rather than sampling error. The null hypothesis is that all studies used to calculate each effect, shared the same effect size. Under the null hypothesis, *Q* should follow a central chi square distribution with degrees of freedom equal to *k* − 1. When the *p* value is less than 0.05, the null hypothesis is rejected, and it can be concluded that there is true variance in the studies' common effect size. ^139^

The random-effects model which assumes that variance between effects is basically random, was applied, and therefore any variance was not attributed to specific moderators. Forest plots, which depicts the effects on a single figure, and also the aggregated effect and its confidence interval, were produced.

Seven separate meta-analysis were performed:

• Meat consumption and mortality
• Plant-based nutrition and mortality
• High fiber diet and mortality
• Plant-based nutrition and diabetes
• Vegetarian/Mediterranean diet and heart disease
• IGF-1 in dairy products and cancer
• Meat consumption and diabetes

The results were calculated using NCSS 2019 software which has a statistical package for calculating aggregated hazard ratio (HR). The input included individual HR from each study, variance for each HR (calculated as SE ^2^, and SE (standard error that was calculated manually using CI with the following calculation:

SE=HR−lower border ofCI÷2

Outputs included the following figures:

1. A forest plot which shows individual HR and its CI, in addition to aggregated HR and CI.
2. A radial plot which shows the study bias for aggregated HR according to heterogeneity.

**Figure 1.**
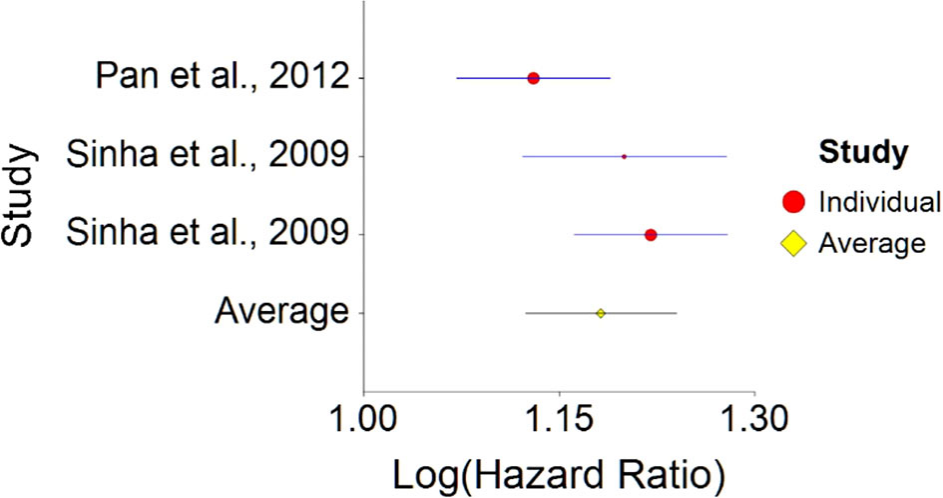
Forest plot of HR between meat consumption and mortality.

**Figure 2.**
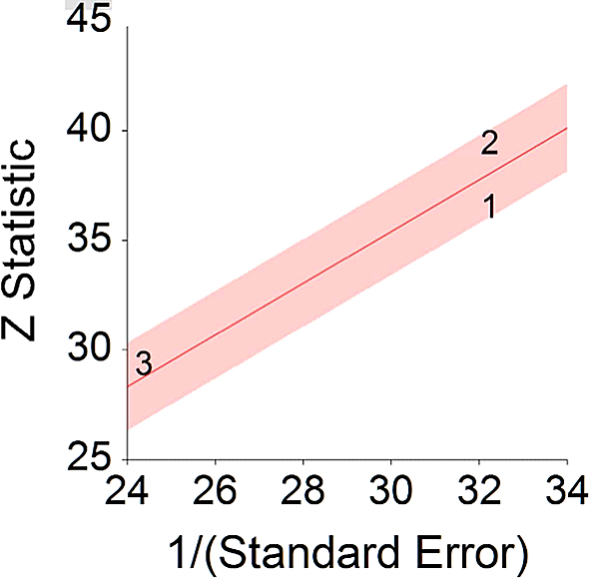
Radial plot of HR between meat consumption and mortality.

**Figure 3.**
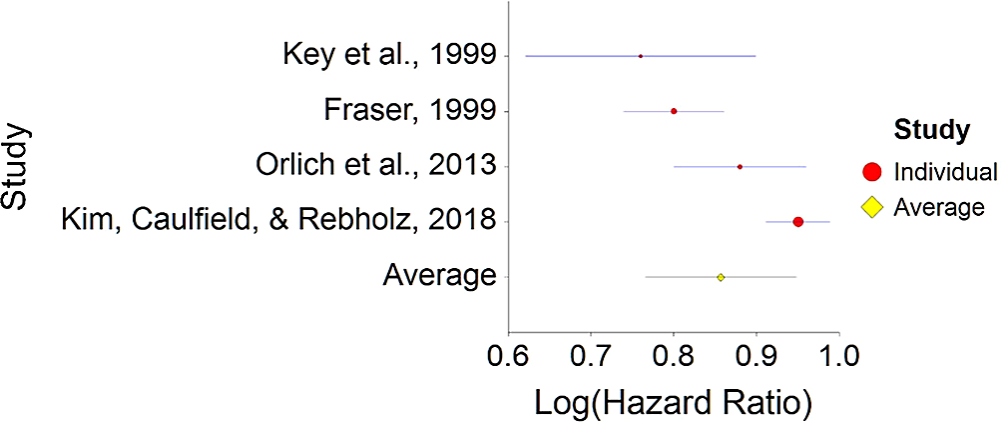
Forrest plot of HR between plant-based nutrition and mortality.

**Figure 4.**
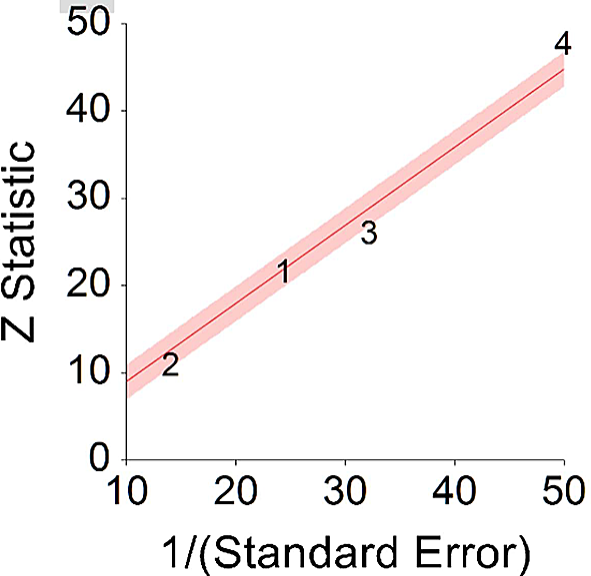
Radial plot of HR between plant-based nutrition and mortality.

**Figure 5.**
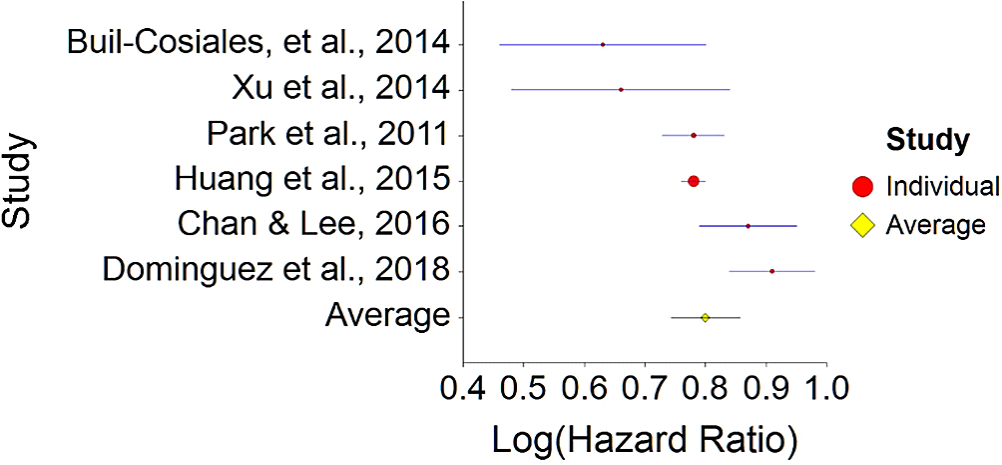
Forest plot of HR between a high fiber diet and mortality.

**Figure 6.**
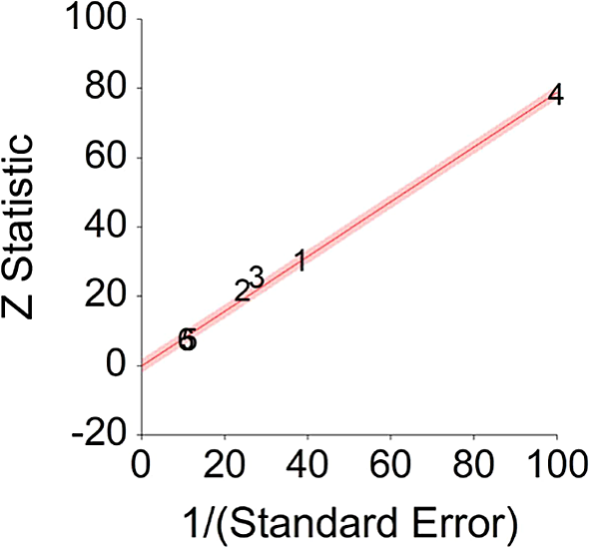
Radial plot of HR between a high fiber diet and mortality.

**Figure 7.**
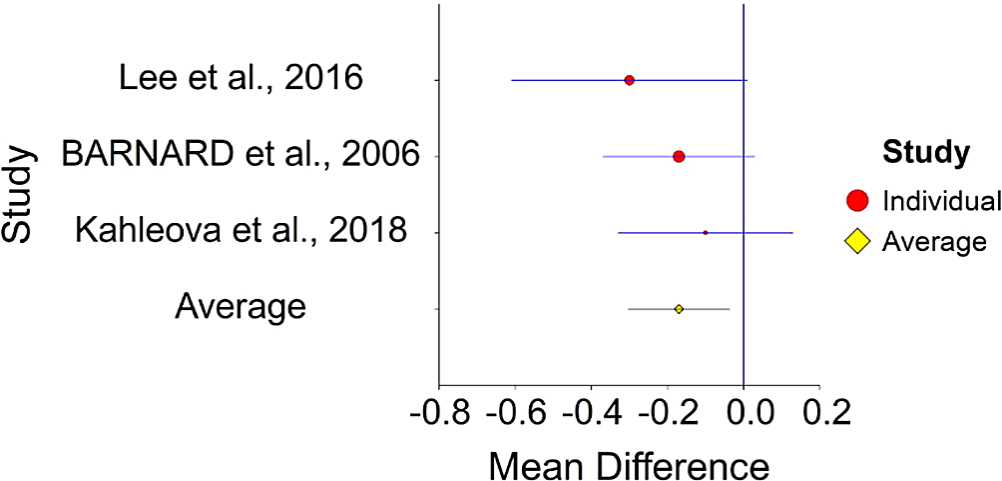
Forest plot of HR between plant-based nutrition and diabetes.

**Figure 8.**
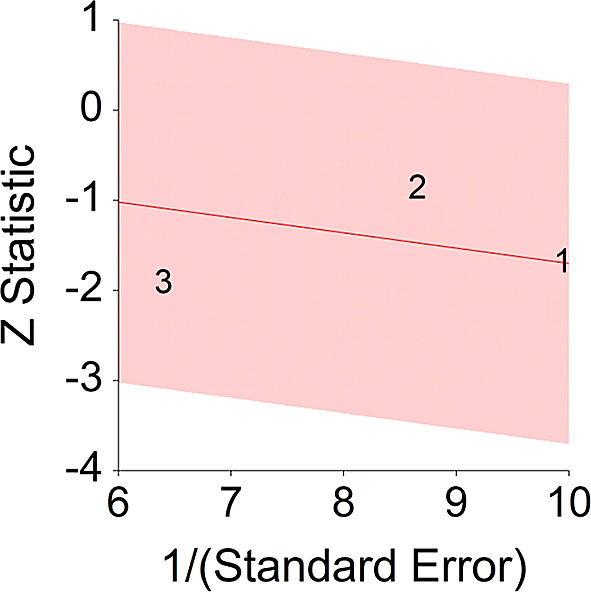
Radial plot of HR between plant-based nutrition and diabetes.

**Figure 9.**
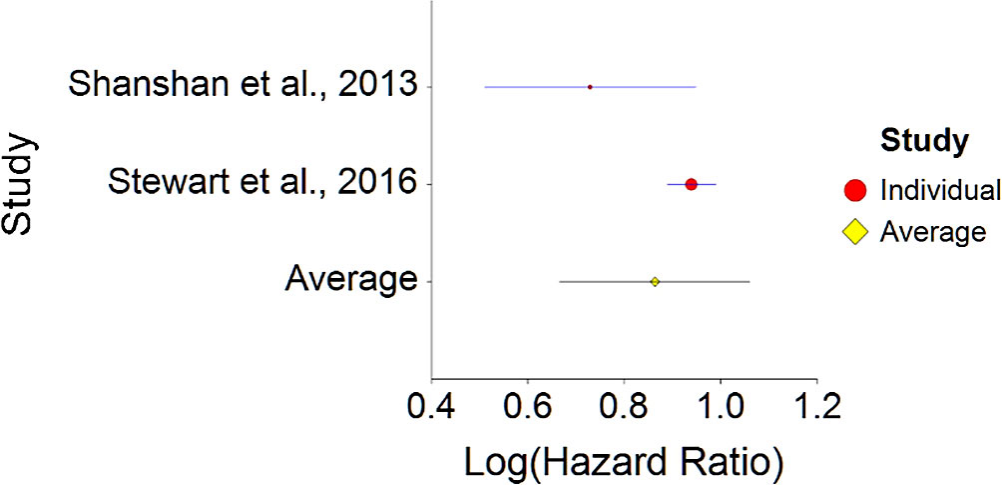
Forest plot of HR between vegetarian or Mediterranean diet and heart disease.

**Figure 10.**
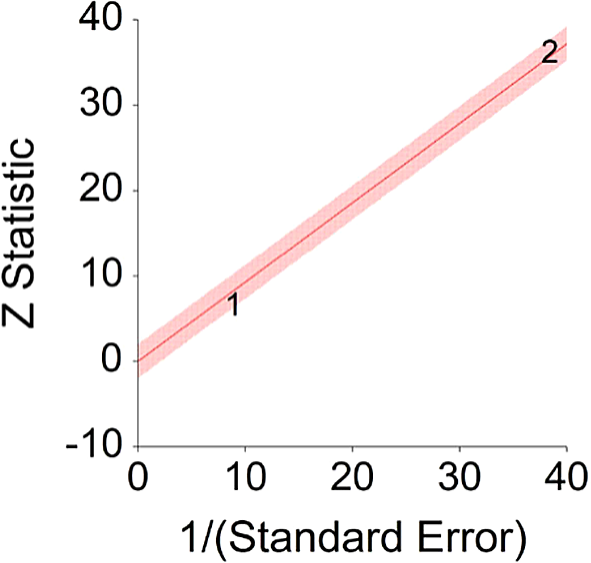
Radial plot of HR between vegetarian or Mediterranean diet and heart disease.

**Figure 11.**
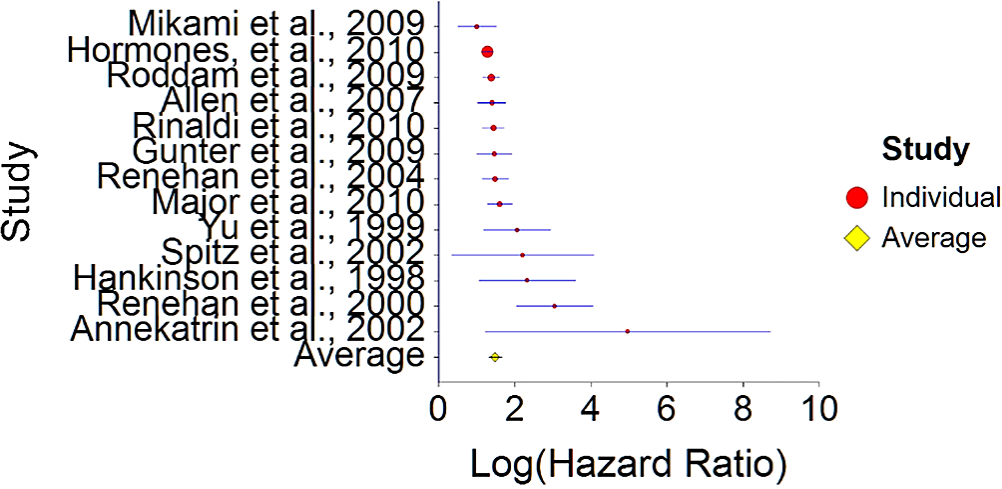
Forest plot of HR between IGF-1 and cancer.

**Figure 12.**
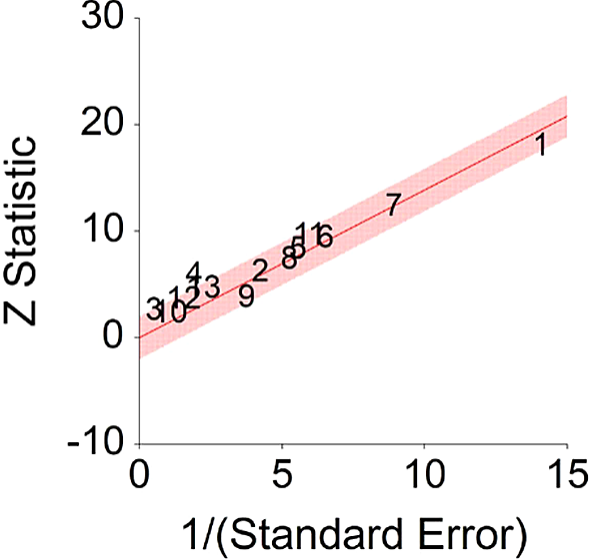
Radial plot of HR between IGF-1 and cancer.

The results are as follows:

1. Meat Consumption and Mortality
  To assess aggregative Hazard Ratio (HR) of meat consumption and mortality, two individual studies were used yielding three effect sizes (see Table 1).
  Results of meta-analysis (n = 666,995) showed that the aggregated effect size between meat consumption and mortality is *HR* = 1.18 (HR S.E. = 0.03, 95% CI [1.12,1.24]) (see Figure 1). This result is significant χ ^2^ (DF = 2) = 3737.16, *p* < 0.001 and means that people consuming meat at any time point during the study period were 18% more likely to die than people that were not consuming meat, and we are 95% confident that people consuming meat are between 12% and 24% more likely to die at any given age than people not consuming meat.
  No heterogeneity was found between studies, meaning there are no potential moderators which could bias this effect, *Q* (2) = 3.80, *p =* 0.09. Hence, differences between individual studies are not significant and considered as homogeneous (see Figure 2, all studies are within the CI borders).
  To conclude, consuming red meat significantly increases death probability by about 20% on average in comparison with not consuming red meat. This effect size is larger compared with effect size received by Wang et al. ^142^ (HR = 1.15). In addition, it is important to note that in a similar meta-analysis conducted by Larsson and Orsini, ^143^ no significant consistent effect was found between meat consumption and mortality.
2. Plant-Based Nutrition and Mortality
  To assess aggregative Hazard Ratio (HR) of plant-based nutrition and mortality, four individual studies were used yielding four effect sizes (see Table 2).
  Results of the meta-analysis (n = 218,712 people) showed that the aggregated effect size between plant-based nutrition and mortality is *HR* = 0.85 (HR S.E. = 0.04, 95% CI [0.77,0.94]) (see Figure 3). This result is significant χ ^2^ (4) = 3497.7, *p* < 0.001 and means that people consuming a plant-based diet at any time point during the study periods were 15% less likely to die than people that were not consuming a plant-based diet, and we are 95% confident that the true value is lying between 6%-23% (we are 95% sure that people consuming a plant-based diet are between 6% and 23% less likely to die at any period of time than people not consuming a plant-based diet).
  A significant heterogeneity was found between studies, meaning that the studies included in this analysis were different by several methodological aspects which could bias the aggregated HR effect, *Q* (3) = 20.70, *p <* 0.01. Differences between individual studies are significant and considered heterogeneous (see Figure 4, result of 4 - Kim, Caulfield, & Rebholz, ^147^ exceeds CI borders), in this study among Seventh-day Adventists, vegetarians were healthier than non-vegetarians but this cannot be ascribed only to the absence of meat.
  To conclude, plant-based nutrition significantly decreases death probability by about 15% on average, in comparison with non-plant-based nutrition.
3. High Fiber Diet and Mortality
  To assess aggregative Hazard Ratio (HR) of a high fiber diet and mortality, six individual studies were used yielding six effect sizes. In addition, two meta-analyses were gathered in order to compare aggregated HR derived from our analysis. These meta-analyses were documented in order to compare results between independent meta-analysis and published meta-analyses (see Table 3).
  Results of meta-analysis (n = 978,380) showed that the aggregated effect size between fiber diet and mortality is *HR* = 0.80 (HR S.E. = 0.03, 95% CI [0.74,0.86]). See Figure 5). This result is significant χ ^2^ (5) = 8155.70, *p* < 0.001. and means that people consuming a high fiber diet at any time point during the study period were 20% less likely to die than people that were not consuming a high fiber diet, and we are 95% confident that the true value is lying between 14%-26% (we are 95% sure that people not consuming meat are between 14% and 26% less likely to die at a given age than people consuming a high fiber diet).
  A significant heterogeneity was found between studies, meaning that the studies included in this analysis are different by several methodological aspects which could bias the aggregated effect, *Q* (5) = 21.44, *p <* 0.01. Hence, differences between individual studies are significant and considered heterogeneous (see Figure 6, result of 3. Dominguez et al., ^150^ exceeds CI borders).
  To conclude, a high fiber diet significantly decreases death probability by about 20% on average, in comparison with non-fiber diet. This effect size is in line with effect size received by Yang et al. ^155^ (HR = 0.84), and by Kim & Je ^154^ (HR = 0.77).
4. Plant-Based Nutrition and Diabetes
  To assess aggregative effect size of plant-based nutrition and diabetes, three individual studies were used yielding three effect sizes. These effects were based on random control trial designs in which individuals in a diet group were compared to individuals in a control group. These designs yielded effect size of difference between means. In addition, a single meta-analysis was found which computed Hazard Ratio between plant-based nutrition and diabetes (see Table 4).
  Results of meta-analysis (n = 133) showed that the aggregated effect size between plant-based nutrition and diabetes is *Cohen’s d* = −0.17 (S.E. = 0.06, 95% CI [−0.30, −0.03]) (see Figure 7). When translated to HR = 0.73 (95% CI [0.58, 0.94]. This result is significant χ ^2^ (2) = 6.24, *p* < 0.05 and means that people consuming a plant-based diet at the end of the trial (dietary change to PBD) showed 27% improvement in their diabetic status.
  No heterogeneity was found between studies, meaning there are no potential moderators which could bias this effect, *Q* (2) = 1.06, *p =* 0.50. Hence, differences between individual studies are not significant and considered as homogeneous (see Figure 8, all studies are within the CI borders).
  To conclude, individuals who keep plant-based have decreased risk of diabetes in comparison with individuals who do not keep this type of diet. This result is stronger in when translated to HR = 0.73, in comparison with effect size for of 0.51. ^159^
5. Vegetarian or Mediterranean Diet and Heart Disease
  To assess aggregative Hazard Ratio (HR) of Healthy diet and heart disease, two individual studies were used yielding two effect sizes. In addition, a single meta-analysis examining this effect was found (see Table 5).
  Results of meta-analysis (n = 19,580) showed that the aggregated effect size between vegetarian or Mediterranean diet and heart disease is *HR* = 0.86 (HR S.E. = 0.10, 95% CI [0.67,1.06]) (see Figure 9). This result is not significant χ ^2^ (1) = 25.10, *p* = 0.413.
  These effects were homogenous between two studies, meaning, studies included in this analysis were not different by methodological aspects which could bias the aggregated effect, *Q* (1) = 3.34, *p =* 0.07 (see Figure 10).
  To conclude, a vegetarian or Mediterranean diet was not found to decrease probability for heart disease. Although non-significant, effect size found in this meta-analysis is similar to effect size received by Kwok et al. ^162^ (HR = 0.84). They come to the conclusion that there is modest cardiovascular benefit, but no clear reduction in overall mortality associated with a vegetarian diet.
  A vegetarian diet can be healthy or not, depending on the foods consumed in this diet. A vegetarian diet that is rich in processed foods, or a Mediterranean diet that has high quantities of meat and dairy products will not produce the health effects desired. Furthermore, only two studies were found that met all the criteria for inclusion.
6. IGF-1 and Cancer
  Insulin-like growth factor 1 (IGF-1) is a protein produced in the liver, encoded by the IGF-1 gene which stimulates growth in cells throughout the body. Protein intake increases IGF-1 levels in humans under age 65, independent of total calorie consumption.
  IGF-1 has a role in regulating lifespan by controlling longevity in mammals and resisting oxidative stress, a known determinant of aging. IGF-1 also plays a role in assisting growth hormones in their anabolic function. It plays several roles in human physiology including tissue growth and development, especially at a young age where it promotes growth in children and ensures that they grow tall. ^163^ IGF-1 is also found in breast milk.
  Research shows that IGF-1 continues to have anabolic effects as the person gets older where increased levels of IGF-1 seem to have several adverse effects on health, as people reach adulthood and age.
  Studies have implicated IGF-1 with a few forms of cancer including colon, pancreas, endometrium, prostate, breast, lung, and colorectal cancer, ^164^^-^^174^ as IGF-1 exerts strong mitogenic actions and triggers a signaling cascade leading to increased proliferation and differentiation of cells and has an anti-apoptotic effect. Certain drug companies are working on medications that reduce the level of IGF-1 in a means to protect from cancer. ^175^ However, there is no definitive association between IGF-1 and cancer in the Japanese population. ^183^^,^^184^ This may be due to the fact that IGF-1 which can also be attained through the diet, is not found in foods regularly consumed as part of the Japanese diet. When examining dairy products and the Japanese population, we will see the same results as with the rest of the population. ^185^
  Epidemiological evidence shows that dairy food consumption significantly increases circulating IGF-1 levels, and dairy consumption after the weaning period maintains high levels of IGF-1 signaling. ^176^^-^^181^ A study showed that when insulin-like growth factor-1 is taken in through the diet, further to the added exogenous dose of IGF-1 in the body, there is also increased stimulation of IGF-1 production in the body, ^182^ which promotes the proliferation of certain cancers.
  To assess the aggregative Hazard Ratio (HR) of dairy products (IGF-1) and cancer, thirteen individual studies were used yielding thirteen effect sizes. ^164^^,^^166^^-^^169^^,^^171^^,^^181^^,^^183^^,^^186^^-^^190^ In addition, a single meta-analysis examining this effect was found (see Table 6). ^191^
  Results of meta-analysis (n = 26,909) of these studies showed that the aggregated effect size between IGF-1 and cancer is *HR* = 1.48 (HR S.E. = 0.09, 95% CI [1.31,1.65]) (see Figure 11). This result is significant χ ^2^ (12) = 914.23, *p* < 0.001 and means that people consuming high IGF-1 products (dairy products) at any time point during the study period were 48% more likely to be diagnosed with cancer than people that were not consuming a high IGF-1 diet, and we are 95% confident that the true value is lying between 31%-65% (we are 95% sure that people consuming dairy are between 31% and 65% more likely to be diagnosed with cancer than people consuming a low/no dairy diet).
  A significant heterogeneity was found between studies, meaning that the studies included in this analysis are different by several methodological aspects which could bias the aggregated effect, *Q* (12) = 25.67, *p <* 0.01. Hence, differences between individual studies are significant and considered heterogeneous (see Figures 11 and 12, result of Annekatrin et al., ^166^ exceeds CI borders).
  To conclude, high IGF-1 levels were found to increase probability for cancer diagnosis by about 48% in comparison with patients with low IGF-1 levels. This finding was larger in comparison with effect size derived from the meta-analysis of Shi et al. ^191^ (HR = 1.05). This suggests that reduced dairy product consumption will lead improved health in the long term.

**Table 1.** Individual studies evaluating HR between meat consumption and mortality.

Study	Total sample	Total death cases	Follow-up (years)	HR	HR 95% CI - Lower	HR 95% CI - Upper	Weight in meta-analysis
1. Pan et al., 2012 ^140^	121,342	23,926	28	1.13	1.07	1.2	36.11
2. Sinha et al., 2009 (men) ^141^	322,263	47,976	10	1.22	1.16	1.29	36.11
3. Sinha et al., 2009 (women) ^141^	223,390	23,276	10	1.20	1.12	1.3	27.79
Total	666,995	95,178		1.18			
**Meta analyses**							
4. Wang et al., 2015 ^142^	1,493,646	150,328		1.15	1.11	1.18	
5. Larsson & Orsini, 2013 ^143^	1,320,980	135,601		1.10	0.98	1.22	

*Note*: HR was calculated for meat consumption vs. non-meat consumption.

**Table 2.** Individual studies evaluating HR between plant-based nutrition and mortality.

Study	Total sample	Total death cases	Follow-up (years)	HR	HR 95% CI - Lower	HR 95% CI - Upper	Weight in meta-analysis
1. Orlich et al., 2013 ^144^	96,469	2,570	5	0.88	0.80	0.97	25.09
2. Key et al., 1999 ^145^	76,172	8,330	10	0.76	0.62	0.94	18.05
3. Fraser, 1999 ^146^	34,192	-	6	0.80	0.74	0.87	27.37
4. Kim, Caulfield, & Rebholz, 2018 ^147^	11,879	2,228	6	0.95	0.91	0.98	29.47
Total	218,712	13,128					

*Note*: HR was calculated for plant-based nutrition vs. non-plant-based nutrition.

**Table 3.** Individual studies evaluating HR between fiber diet and mortality.

Study	Total sample	Total death cases	Follow-up (years)	HR	HR 95% CI - Lower	HR 95% CI - Upper	Weight in meta-analysis
1. Park et al., 2011 ^148^	567,169	31,456	9	0.78	0.73	0.82	22.14
2. Chan & Lee, 2016 ^149^	15,740	3,164	6	0.87	0.79	0.97	17.54
3. Dominguez et al., 2018 ^150^	19,703	323	10.1	0.91	0.84	0.99	19.06
4. Huang et al., 2015 ^151^	367,442	46,067	14	0.78	0.76	0.80	26.07
5. Buil-Cosiales, et al., 2014 ^152^	7,216	425	8.7	0.63	0.46	0.86	7.90
6. Xu et al., 2014 ^153^	1,110	300	10	0.66	0.48	0.91	7.29
Total	978,380	81,735					
**Meta analyses**							
7. Kim & Je, 2016 ^154^	1,409,014	45,078	-	0.77	0.71	0.84	-
8. Yang et al., 2015 ^155^	982,411	67,260	-	0.84	0.80	0.87	-

*Note*: HR was calculated for fiber diet vs. non-fiber diet.

**Table 4.** Individual studies evaluating Effect size between plant-based nutrition and diabetes.

Study	Cases in vegan diet	Cases in non-vegan diet	Cohen’s d	95% CI - Lower	95% CI - Upper	Weight in meta-analysis
1. BARNARD et al., 2006 ^156^	21/49	13/50	−0.32	−0.57	−0.07	
2. Kahleova et al., 2018 ^157^	38	37	−1.0	−1.2	−0.8	
3. Lee et al., 2016 ^158^	46 −0.5 SD 0.8	47 −0.2 SD 0.7	−0.40	−0.65	−0.15	
Total	133	134				

Meta analyses	Total sample	Total diabetes cases	HR	HR 95% CI – Lower	HR 95% CI - Upper	-
4. Tonstad et al., 2009 ^159^	60,903	3,430	0.51	0.40	0.66	

**Table 5.** Individual studies evaluating HR between vegetarian or Mediterranean diet and heart disease.

Study	Total sample	Total heart disease cases	Follow-up (years)	HR	HR 95% CI - Lower	HR 95% CI - Upper	Weight in meta-analysis
1. Li et al., 2013 ^160^	4,098	1,133	-	0.73	0.51	1.04	36.54
2. Stewart et al., 2016 ^161^	15,482	2,885	3.7	0.94	0.89	0.99	64.36
Total	19,580	4,018		1.18			
**Meta analyses**							
3. Kwok et al., 2014 ^162^	183,321	-	-	0.84	0.74	0.96	-

*Note*: HR was calculated for vegetarian or Mediterranean diet vs. non healthy diet.

**Table 6.** Individual studies evaluating HR between IGF-1 and cancer.

Study	Control	Cancer cases	HR	HR 95% CI - Lower	HR 95% CI - Upper	Weight in meta-analysis
1. Endogenous Hormones, T. E., & Breast Cancer Collaborative Group, 2010 ^190^	9,428	4,790	1.28	1.14	1.44	17.28
2. Gunter et al., 2009 ^189^	841	810	1.46	1.00	2.13	8.09
3. Annekatrin et al., 2002 ^166^	263	132	4.97	1.22	20.2	8.72
4. Renehan et al., 2000 ^164^	293	52	3.05	2.04	4.57	0.21
5. Renehan et al., 2004 ^167^	7137	3609	1.49	1.14	1.95	2.51
6. Rinaldi et al., 2010 ^171^	1121	1121	1.43	1.13	1.93	10.72
7. Roddam et al., 2008 ^169^	5200	3700	1.38	1.16	1.60	12.21
8. Allen et al., 2007 ^168^	630	630	1.39	1.02	1.89	14.77
9. Mikami et al., 2009 ^183^	302	101	1.01	0.49	2.10	10.2
10. Spitz et al., 2002 ^188^	297	297	2.21	0.35	12.84	6.98
11. Major et al., 2010 ^181^	559	74	1.61	1.28	2.02	5.13
12. Hankinson et al., 1998 ^186^	620	397	2.33	1.06	5.16	3.23
13. Yu et al., 1999 ^187^	218	204	2.06	1.19	3.56	17.28
Total	26,909	15,917				
**Meta analyses**						
14. Shi et al., 2004 ^191^	6030-1017	30-397	1.05	0.94	1.17	

*Note*: HR was calculated for cancer vs. non cancer.

### Meat consumption and diabetes

To assess aggregative Hazard Ratio (HR) of meat consumption and diabetes, two individual studies were used yielding 3 effect sizes (see Table 7).

**Table 7.** Individual studies evaluating HR between meat consumption and diabetes.

Study	Total sample	Total Diabetes cases	Follow-up (years)	HR	HR 95% CI - Lower	HR 95% CI - Upper	Weight in meta-analysis
1. Pan et al., 2013 ^217^	149,143	7,540	16-20	1.99	1.53	2.58	46.38
2. Pan et al., 2011 ^216^	204,157	13,759	-	1.12	1.08	1.16	53.62
Total	353,300	21,299					
**Meta analyses**							
3. Aune, Ursin & Veierod, 2009 ^218^				1.17	0.92	1.48	
4. Micha et al., 2011 ^219^	1,218,380	10,797	-	1.16	0.92	1.46	-

*Note*: HR was calculated for meat-consumption vs. non-meat-consumption.

**Figure 13.**
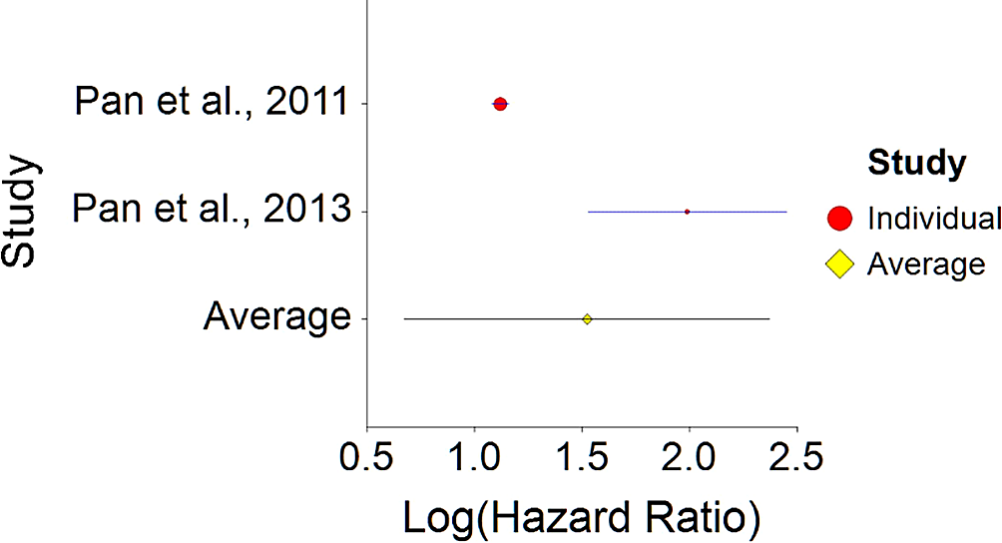
Forrest plot of HR between meat consumption and diabetes.

Results of meta-analysis of these studies (n = 353,300) showed that the aggregated effect size between meat consumption and diabetes is *HR* = 1.52 (HR S.E. = 0.43, 95% CI [1.17, 2.37]) (see Figure 13). This result is significant χ ^2^ (1) = 3194.10, *p* < 0.001.

**Figure 14.**
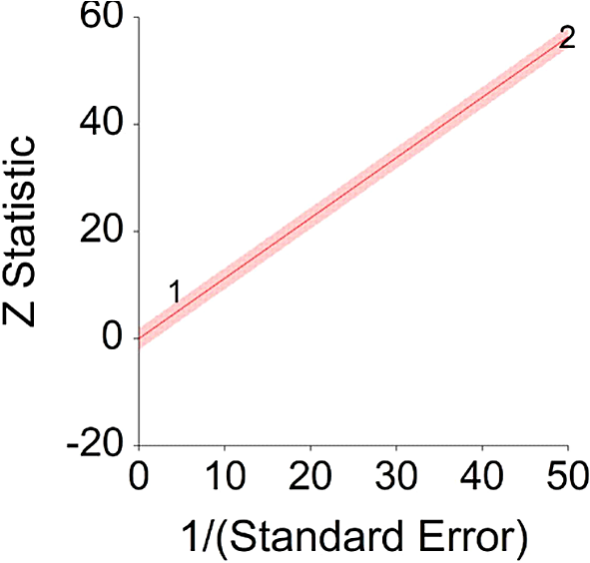
Radial plot of HR between meat consumption and diabetes.

A significant heterogeneity was found between two studies, meaning, studies included in this analysis are different by several methodological aspects which could bias the aggregated effect, *Q* (1) = 13.61, *p <* 0.01 (see Figure 14, results of Pan et al., ^216^ exceeds CI borders).

To conclude, meat consumption significantly increases probability for diabetes by about 50% on average, in comparison with vegetarian nutrition. This effect size is larger in comparison with effect size received by Aune et al. ^218^ (HR = 1.17), and by Micha et al. ^219^ (HR = 1.16).

## Results

The results from these meta-analyses presented, which involved 2,264,009 people (9,600,738 including previous meta-analyses for which there may be overlap), of which 220,906 (734,711 including previous meta-analyses for which there may be overlap) became sick or died during the studies, aimed to assess aggregate effect sizes of several nutrition types with both mortality and diseases.

To conclude, all meta-analyses conducted to assess mortality showed highly significant results. Specifically, meat consumption increased mortality probability by 18% on average, a plant-based diet and a high fiber diet decreased mortality in 15% and 20% respectively. In addition, dietary diary consumption (as measured by IGF-1) was found to increase probability for cancer by about 48%, while plant-based nutrition reduced diabetes by about 27% and meat consumption increased probability for diabetes by about 50% on average. No significant effects were indicated for meat consumption or vegetarian or Mediterranean diet on diabetes or heart disease (see Table 8).

**Table 8.** Aggregate Effect Sizes for Meta-Analyses.

	Aggregate effect size	95% CI - Lower	95% CI - Upper	Significant
**Mortality**				
Meat consumption and mortality	1.18	1.12	1.24	Yes
Plant-based nutrition and mortality	0.85	0.77	0.95	Yes
High fiber diet and mortality	0.80	0.74	0.86	Yes
**Disease**				
Vegetarian/Mediterranean diet and heart disease	0.86	0.67	1.06	No
IGF1 and cancer	1.48	1.31	1.65	Yes
Plant-based nutrition and diabetes	0.73	0.58	0.94	Yes
Meat consumption and diabetes	1.52	1.17	2.37	Yes

The possible limitations of these meta-analysis should be taken into consideration. Although the combination of results from different studies will increase statistical power in detecting significant associations because of the increased sample size, however, this often results in heterogeneity. Heterogeneity is expected as the studies took place in different geographic locations, used different dietary assessment methods, and included participants who are in different gender and age groups. In general, there was significant heterogeneity in many of the meta-analyses, as can be seen in the redial plots.

Publication bias is another concern. The statistical tests did not suggest the presence of publication bias in these meta-analyses, although some may have had limited statistical power due to the sometime low number of studies, but on the other hand, very large numbers of participants do reduce this bias.

Completely ruling out the possibility of residual confounding or a temporal bias cannot be done, but if the associations found are real, then it is safe to say that a whole food plant-based diet can reduce the risk for common diseases and increase longevity.

### Summary

The evidence from this research component suggests that the most suitable diet for human consumption for health and longevity is a natural, whole food, high-fiber and 90+% plant-based diet, with small amounts of lean meat. This diet leads to health for our species, reducing the need for and costs of healthcare, especially for the growing elderly population.

In order to produce change and have more of the population follow this type of diet, governments and individuals need practical methods and health policies that improve health, with a smaller carbon footprint.

The consumption of animal products not only influences personal health, but also environmental health. The current situation shows that the rearing of livestock and meat consumption on a commodity-basis, accounts for the highest greenhouse gas (GHG) emissions, respectively, producing 41% and 20% of the sector’s overall GHG output. ^192^ Rearing of livestock is also the single greatest anthropogenic source of methane, a GHG about 25 times more powerful at trapping heat in the atmosphere than CO _2_, (from raising cattle for food) and nitrous oxide emissions (from fertilizer and manure usage), two very potent GHGs.

Rearing of livestock is responsible for approximately 37% of anthropogenic methane emissions and approximately 65% of human nitrous oxide emissions globally. ^193^

Furthermore, animal agriculture is a notable contributor to global warming due to the quantities of fossil fuels used, together with deforestation. Worldwide, energy from fossil fuels are responsible for 40% of human GHG emissions, which does not include deforestation at about 18%, and animal agriculture 18%. In fact, most deforestation is done for the purpose of rearing animals and to expand pastures and arable land used to grow crops for feeding livestock. Thus, of the 91-97% human induced GHG emissions, 60% is due to animal agriculture.

Manufacturing beef demands significantly higher resources. Beef production needs 28 times more land, six times more fertilizer and 11 times more water than the production of chicken or pork. Furthermore, producing beef releases four times more GHGs than the same amount of pork on a calorie basis, and five times more than poultry. ^194^

The consumption of plant-based foods produces very low GHG emissions. ^195^ It is more economical to grow crops for food than to grow crops for animal feed, necessitating the build-up of muscle mass and bone tissue.

Overall, the goal of agriculture and governments must be to build a sustainable future and to support the health of the population. This means finding solutions that will continue to meet human food and energy requirements in cheap, safe, and high-quality ways even for a growing human population, while leaving little or no negative effects on our planet, along with disease control and caring for animal welfare, in a way that is profitable for the farmer.

### Policies for change

To ensure that every person on the planet would be able to meet their nutritional needs in the future, we would need to (1) build stable national relationships between different countries for consistent import and export of agricultural goods for food security, (2) establish domestic and global policies including meat and dairy taxes to be implemented to ensure that a price is paid for the destruction of the earth and its resources, and (3) make agricultural policies and trade rules compatible with global food security and sustainability.

We also need to improve dietary habits. The number of men and women existing in sub-optimum health is a dramatic 95% of the global population, and this number is estimated to grow in the upcoming years. ^196^^-^^198^

If there is no change in prevailing dietary habit trends, GHG emissions in 2050 connected with food systems will rise by 51% compared with current levels.

If the global population followed a 90% plant-based diet, with meat and animal products forming 10% of the diet, GHG emissions would decrease by 55%. These statistics show a clear and simple way to effect change. By reducing meat and dairy products from the diet to twice a week and changing the composition of the common diet to mostly vegetables, nuts, fruits, whole grains, nuts and legumes, we can immensely influence global GHG emissions, slow deforestation, and prevent many diseases.

Governments and civil society are profoundly unwilling of to intrude into people’s diets and tell them what to eat. But we will soon understand that this is the only way to go.

Although reducing overall meat and dairy product consumption will help, the type of meat chosen also has an immense effect on our planet and the future of food. Some animal products are more sustainably produced than others. A way of comparing species is to look at how efficiently an animal converts feed into biomass for human nutrition.

One needs only 1.1 kg of pellet food to get 1 kg of fish meat. ^199^^-^^201^

Chickens also use feed efficiently, where 1.7 kg of feed produces 1 kg of chicken because they are grown quickly and slaughtered at a young age.

By comparison, 2.9 kg of feed is needed for the making of 1 kg of pig meat, and 6.8 kg of feed is needed for the making of 1 kg of cow meat.

A reduction in animal consumption would have major effects on life on Earth:

• Of the available 12 billion acres of agricultural land available on earth, 68% is currently used for livestock. ^202^ Some of this land could be restored for grasslands and forests, to help capture carbon further reducing carbon emissions or be diverted to growing plants for human consumption.
• People previously involved in the livestock industry, (about one-seventh of the global population), would require help making the shift towards a different career, whether in plant-based agriculture, in reforestation, in the biofuel industry from the byproducts of crops now used as food for livestock, or in caring for the animals (re) introduced into the wild or into sanctuaries and zoos.
• About one-third of the planet's land is arid to semi-arid rangeland only able to support livestock agriculture. In these areas, land could be used to house the growing African population, for vertical farming facilities, for growing native trees found to be of medicinal value (e.g., moringa or shea) and for growing livestock for wool for populations such as the Mongols and Berbers, who would otherwise lose their cultural identity, causing them to settle permanently in cities or towns. Solar farms could also be located on this land, providing sustainable sources of energy to local communities.

Apart from the myriad reasons to lower meat consumption, meat has an important role in tradition and cultural identity. Giving up meat has impact on the culture of many societies, so governments and people have personally failed to reduce meat consumption.

This indifference can be combated by increasing the price of meat so that farmers can raise fewer animals and earn the same. This shift in production would make meat take the form of a treat rather than a staple food, as it is today.

Governments can subsidize fresh vegetables and fruits, making them more affordable and more widely accessible to all populations, instead of subsidizing meat and dairy products.

The environmental issue may also be solved with meat coming from the use of technology, such as lab-grown meat and fish. ^203^^-^^205^

The current problem is that newly rich societies are increasing their demand for animal products. As people’s incomes increase, they start buying more dairy, poultry and meat and fish.

Therefore, to improve people’s eating habits, a whole food mostly plant-based diet should be encouraged and taught in schools including medical school. Just as teaching first aid is common practice all over the world, the same should be done with regard to plant-based food choices.

## Discussion

As we see, food choices are very dependent on prices, and can therefore be influenced by prices.

Difficulties also arise in making healthy food choices in food swamps, where affordable, fresh and healthy foods *are* accessible, but where there is an overabundance of energy dense, low-nutrient foods as well. Here, unhealthy food choices are much easier to make than healthy food choice, due to their cheaper prices. This is where education is critical.

### Sugar taxes

Since sodas are not a necessary element of a wholesome diet, soda is a welcome candidate for taxation. We see that if a tax of about 20% is introduced, it has a serious effect on the buying behavior of consumers producing many health benefits.

In Mexico, a sugar tax on soft drinks has been successful due to the fact that the funds were spent providing free drinking water in schools. ^206^^-^^208^

### Meat and dairy taxes

The price of animal products must match their real cost to society, including their carbon footprint. A meat tax puts a specific price on the harm they cause the environment. Currently, there are no consequences for the raising of livestock, even though these industries are proven to be detrimental to health and the environment. There must be a financial deterrent such as taxes, fines, or penalties to discourage their production and usage. To date, no economic incentives are in place for industries or individuals to move away from the generation and consumption of animal goods. Taxing meat and dairy products will put economic pressure on people and these industries to make change.

This tax money can then be given back to meat farmers as government support.

People will still buy meat, but more as a treat rather than as a staple.

A meat tax will lead to a major reduction in GHG emissions and preserve over 500,000 lives per year through healthier diets.

If we add a 35% meat and dairy tax and encourage sellers to sell meat and dairy products at 50% higher prices, people will make different choices.

Food stamps:

For 40 years, the food stamps program has been a very significant domestic hunger safety net that helps provide economic well-being, access to proper nutrition, food security and accessibility, and a reduction of child poverty and money for food spending that benefits those most in need. Food stamps are also good for the economy. ^209^

There is one major drawback of such programs, namely the foods they include.

People can use food stamps to purchase any food item for human consumption, including candy, soft drinks, ice-cream, crackers, cookies, and cakes. ^210^

Food stamp policy should change to allow the purchase of only natural foods, without options for foods that are the leading causes of illness and chronic diseases.

Food stamps can thus help guide populations using them towards making healthy food choices by default. In this way, the government will target the poorest, most needy families first and this will lead the way for agriculture to follow suit.

Subsidies

**Figure 15.**
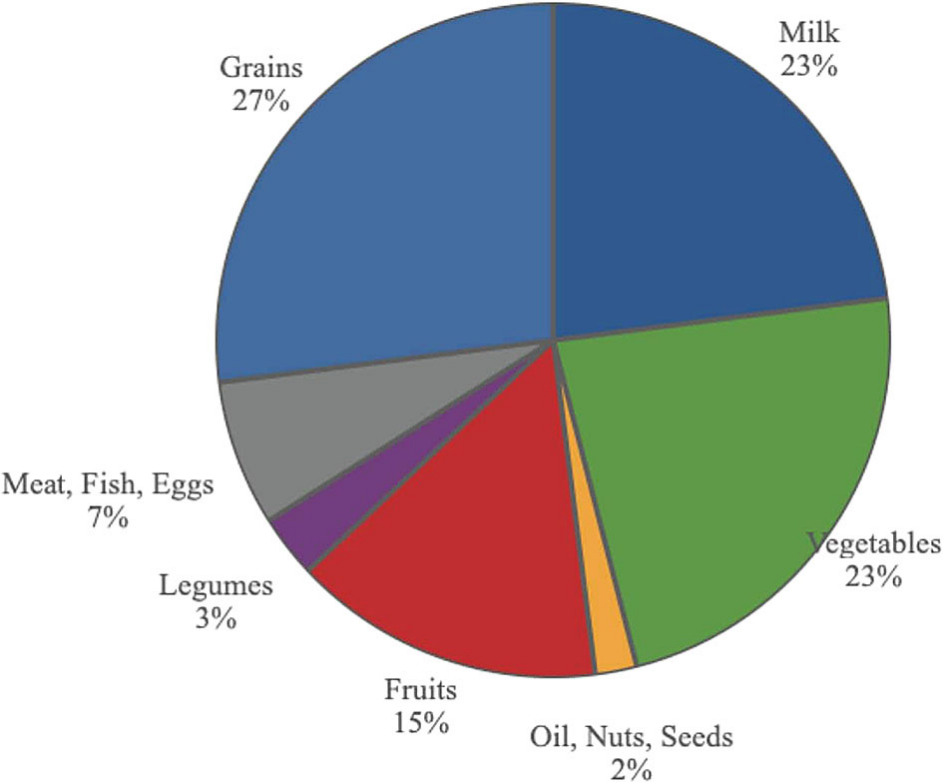
The distribution of foods in the current USDA food pyramid.

In the current US food pyramid, (see Figure 15), the USDA suggests that meat, poultry, fish, eggs and legumes together should comprise 10% of our diet and that dairy products should comprise 23% of our diet. When putting these foods together and reducing intake of legumes, the pyramid suggest we should be consuming about 30% of our calories from animal-based products. ^211^^,^^212^ However, the meat and dairy industries get 74% of USDA subsidies. Vegetables and fruits, according to the USDA food pyramid, are to form 38% of total calories. However, these industries get under 3% of the subsidies. ^213^

Grains receive 13% of subsidies, with most going to feed livestock; sugar, oil, starch and alcohol, 11%; nuts and legumes, 1.9%; and fruits and vegetables, 0.4% of subsidies.

In order to improve health, subsidies should become more similar in percentages to the USDA’s tiers in the food pyramid.

**Figure 16.**
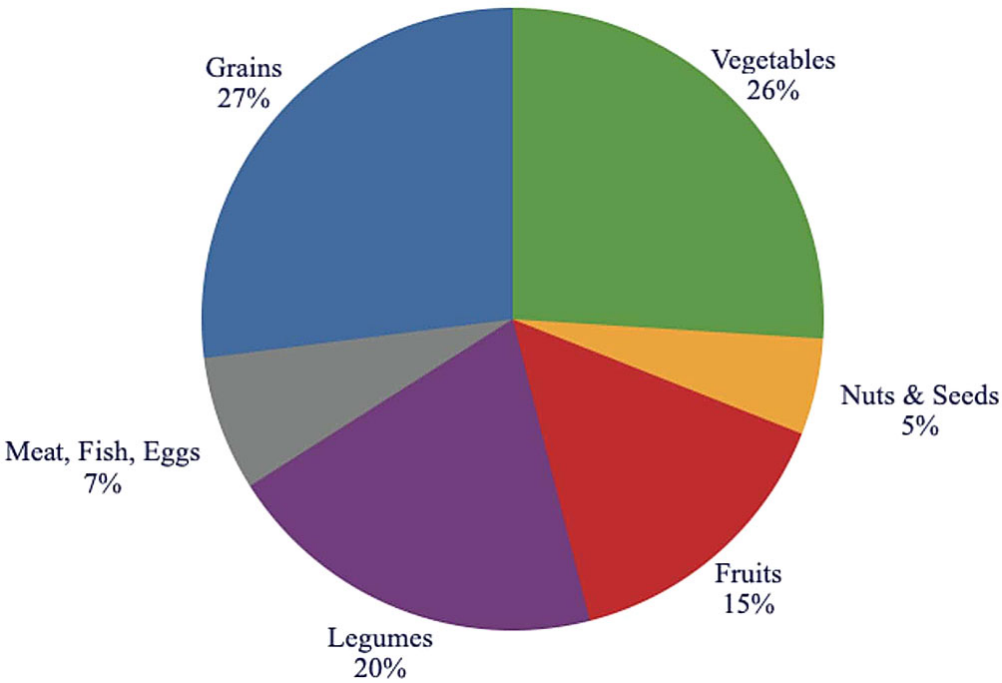
The distribution of foods in the recommended food pyramid.

The Recommended Food Pyramid Based on This Research:

According to this research into the ideal diet for humans the optimal food pyramid would reflect the following breakdown (see Figure 16):

• *grain* consumption (27%), recommending that all grains should be consumed as whole grains.
• *vegetables* (26%), highlighting a variety of dark green vegetables, as well as root vegetables.
• *legumes* (20%), peas, lentils and beans, and their spreads.
• *fruits* (15%), emphasizing variety and deemphasizing fruit juices.
• *meat* (7%), emphasizing lean meats such as fish, and chicken.
• *oils, nuts, seeds* (5%), recommending nuts and seeds and their pastes as sandwich toppings.
• *honey*, emphasizing whole natural honey (0-0.5%).

To conclude, a whole-food, high-fiber, plant-based diet consisting mainly of whole grass grains, legumes, USOs, nuts, seeds and fruits, with reduced quantities of meat and dairy products has been statistically proven to not only prevent most modern-day diseases but may also reverse them while supporting the growing population in a healthy and sustainable way.

Individuals and governments should aim to use this knowledge through policies, to feed the growing global population in a healthy and sustainable way without causing further environmental destruction.

In developed countries, interventions can include increased income-earning opportunities, changes in the food pyramid, meat, dairy, and sugar taxes, a change in food stamp guidelines, subsidies for farmers, support for excess food sharing, supermarket availability for all communities, and school feeding and education programs in all countries. In developing countries, this effort could include local markets for food producers, improved infrastructure, secured purchasing power through governmental prevention of price fluctuations, securing land ownership, easier access to credit, knowledge-sharing through demonstration farms and websites, and legal structures supporting private investors.

## Conclusions

All meta-analyses conducted to assess mortality showed highly significant results. Specifically, meat consumption increased mortality probability by 18% on average, plant-based diets and fiber-rich diets decreased mortality by 15% and 20%, respectively.

In addition, dairy consumption (as measured by IGF1) was found to increase the probability of cancer by about 50%, while plant-based nutrition reduced diabetes by about 27%.

To conclude the findings, a whole-food, high-fiber, plant-based diet consisting mainly of whole grass grains, legumes, USOs, nuts, seeds, and fruits, with reduced quantities of meat and dairy products, has been statistically proven to prevent most modern-day diseases while supporting the growing population healthily and sustainably.

Individuals and governments should use this knowledge and begin the process of change to support, through policies, the feeding of the growing global population healthily and sustainably without causing further environmental destruction. Future research should examine different aspects of whole food plant-based diets and their effects on health and mortality, such as nut and seed consumption and disease. Also, research into dietary recommendation strategies is necessary to translate the findings of this study to influence a wider population.

### Glossary of terms

Z Statistic, standardized score that indicates a higher probability for a significant result; Hazard ratio (log), the ratio of the hazard rates corresponding to the conditions described by two levels of an explanatory variable that explain the outcome in survival analysis; Forest plot, a graphical display of estimated results from a number of scientific studies addressing the same correlation in a meta-analysis. The forest plot depicts the relationship between an independent variable and the outcome across several similar correlations; Radial plot, a graphical display of heterogeneity in the data in a meta-analytic context. For a fixed-effects model, the plot shows the inverse of the standard errors (1/standard error) on the horizontal axis against the observed effect sizes or outcomes standardized by their corresponding standard errors on the vertical axis

## Data availability statement

### Underlying data

Open Science Framework: Manuscript - The Ideal Diet for Humans. https://doi.org/10.17605/OSF.IO/64NAM. ^215^

This project contains the following underlying data:

• Research Findings Galit Goldfarb.pdf (the search strategy and results)

### Reporting guidelines

The reporting of this systematic review was guided by the standards of the Preferred Reporting Items for Systematic Review and Meta-Analysis (PRISMA) Statement openly available in Open Science Framework at https://doi.org/10.17605/OSF.IO/64NAM.

This repository includes the following files:

• PRISMA 2009 flow diagram Galit Goldfarb.pdf
• PRISMA 2009 checklist.pdf

Data are available under the terms of the Creative Commons Attribution 4.0 International license (CC-BY 4.0).
