# Supplementary material for: The Ideal Diet for Humans to Sustainably Feed the Growing Population – Review, Meta-Analyses, and Policies for Change
Source: F1000Res. 2023 Jun 20;10:1135. Originally published 2021 Nov 10. [Version 2] doi: 10.12688/f1000research.73470.2 (PMC10623543; doi:10.12688/f1000research.73470.2)
Supplement: Peer review history

# Reviewer report: Reviewer response for version 2

**DOI:** 10.5256/f1000research.147677.r194278

**Published:** 2023-11-01

**Authors:** Zakira Naureen

**Affiliations:** 1 University of Nizwa, Nizwa, Ad Dakhiliyah ‍Governorate, Oman

This manuscript is a good attempt on collecting the information across the globe to suggest an ideal diet for human. On scientific grounds the paper is sound enough to be accepted. However suggesting an ideal diet for human across the globe is not an easy task at all because the diet humans eat is based on their geographical, socioeconomic, cultural, religious, behavioral norms and besides that it varies from person to person their metabolic and hormonal state, life style pattern etc. 

 As a systematic review this is good job indeed, and I recommend acceptance, however it would have been better if they had classified the data according to geography, and socioeconomic status.

Are the rationale for, and objectives of, the Systematic Review clearly stated?

Yes

Is the statistical analysis and its interpretation appropriate?

Yes

Are sufficient details of the methods and analysis provided to allow replication by others?

Yes

Are the conclusions drawn adequately supported by the results presented in the review?

Yes

Reviewer Expertise:

Metabolomic and genomic studies in plant animals and human. Recently working on genetic and metabolomic factors of obesity.

I confirm that I have read this submission and believe that I have an appropriate level of expertise to confirm that it is of an acceptable scientific standard.

# Reviewer report: Reviewer response for version 1

**DOI:** 10.5256/f1000research.77123.r163444

**Published:** 2023-03-22

**Authors:** Aysha Karim Kiani

**Affiliations:** 1 Department of Biological Sciences, National University of Medical Sciences, Rawalpindi, Punjab, Pakistan; 2 MAGI EUREGIO,, Bolzano, Italy

This review plus meta-analysis has discussed effect of different food components on the quantity/longevity as well as quality of life. Introduction is well written and gives comprehensive picture of evolutionary changes in the dietary habits of man. Although, introduction should also include some information about current dietary habits and type of foods options available such as processed food to give.

 The objectives and rationales of this systemic review are stated but they should be written under proper headings. Methodology is well explained and results are supported by the conclusions of previous studies.

 This review elaborated that more consumption of plant based diet including vegetables, grains and fruits, increases the life span and prevent diseases while the over consumption of meat and dairy product decreases the life span as well as effects the health of people around the world.

 In addition to the facts stated in this review it will be encouraged to also analyze the effect of these food components in relation with the geographical distribution and highlight the difference in the food or caloric requirements among people from different geographical regions. The policies suggested are valid and conclusions drawn are significant to consider.

Are the rationale for, and objectives of, the Systematic Review clearly stated?

Partly

Is the statistical analysis and its interpretation appropriate?

I cannot comment. A qualified statistician is required.

Are sufficient details of the methods and analysis provided to allow replication by others?

Yes

Are the conclusions drawn adequately supported by the results presented in the review?

Yes

Reviewer Expertise:

Human genetics, Molecular Biology, Immunology, Diabetes, nutrigenomics

I confirm that I have read this submission and believe that I have an appropriate level of expertise to confirm that it is of an acceptable scientific standard, however I have significant reservations, as outlined above.

# Reviewer report: Reviewer response for version 1

**DOI:** 10.5256/f1000research.77123.r145030

**Published:** 2022-09-29

**Authors:** Katherine Curi Quinto

**Affiliations:** 1 School of Public Health, Nutrition Research Institute, Cuernavaca, Mexico

1. Complement the introduction with background and rationality regarding the “Current Ideal dietary patterns” The historical track of changes of the human diet, is ok. But what about the current recommended dietary patterns such the one from the EAT Lancet commission, flexitarian diet, etc.?
2. Be clear in the methods section. The first two lines do not contribute with the clarity of the explanation of the methods, I suggest to drop it. 
3. Explain the rationality to make the research using terms regarding food groups instead of looking the impact of a dietary pattern which includes all the components of the diet.
4. Include in the methods the quality control for the selection/identification of the paper included in the review, as well as the quality of the papers itself.  
5. It would be helpful to explain the different dietary patterns included in the review, because you want to recommend an “ideal diet”. Eg. What is the “Plant based diet” or “plant based nutrition” as the author mentions.  
6. In the discussion the authors include some recommendation for policy such as taxes for meat, it would be important to discuss about population that eat low amounts of meat due to low economical access, this population is vulnerable to important nutritional deficiencies that are caused of mortality and morbidity in early years. Remember that the main nutritional problem in developing countries is the malnutrition with the coexistence of excess weight. 
7. Discuss how representative are the review in geographical terms. The results could be transferable to low-income countries. 

Are the rationale for, and objectives of, the Systematic Review clearly stated?

Partly

Is the statistical analysis and its interpretation appropriate?

Yes

Are sufficient details of the methods and analysis provided to allow replication by others?

Partly

Are the conclusions drawn adequately supported by the results presented in the review?

Partly

Reviewer Expertise:

NA

I confirm that I have read this submission and believe that I have an appropriate level of expertise to confirm that it is of an acceptable scientific standard, however I have significant reservations, as outlined above.
